# Red Blood Cell Transfusion and Cardiac Surgery–Associated Acute Kidney Injury∗

**DOI:** 10.1016/j.jacbts.2022.04.011

**Published:** 2022-07-25

**Authors:** Rajesh Gupta

**Affiliations:** Division of Cardiovascular Medicine, Department of Medicine, University of Toledo, Toledo, Ohio, USA

**Keywords:** acute kidney injury, cardiac surgery, packed red blood cells, transfusion, trogocytosis

**Figure:**
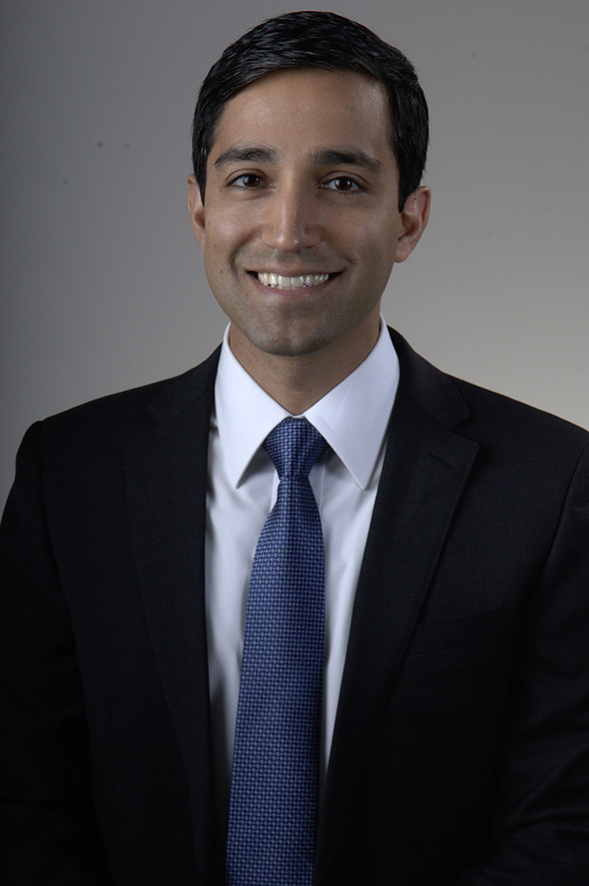

Cardiac surgery is one of the most common major surgeries performed in the United States and globally. Each year, almost 1 million cardiac surgeries are performed in the United States alone. Cardiac surgery–associated acute kidney injury (CSA-AKI) occurs in 20%-30% of patients. CSA-AKI is typically identified by a rising creatinine or poor urine output following cardiac surgery. Any degree of AKI is associated with worse outcomes after cardiac surgery. Furthermore, severe AKI requiring renal replacement therapy is a very powerful predictor of mortality and is associated with an approximately 50% risk of in-hospital mortality.^1^

CSA-AKI has a multifactorial pathogenesis that is incompletely understood. The main purported pathophysiological mechanisms can be grouped into 3 broad categories: ischemia-reperfusion injury, heme toxicity, and inflammation.^1^ Hemodynamic disturbances and changes in renal perfusion occur during and after cardiac surgery. Cardiac surgery involves general anesthesia, aortic cross clamping, vasopressor use, and blood product and intravenous fluid administration, all of which have hemodynamic effects. Cardiac surgery also typically involves stopping the heart. During this time, the body is oxygenated and perfused via a cardiopulmonary bypass (CPB) machine. There may be periods of hypotension or relative underperfusion of the kidneys during this process. Although mean arterial pressure can be tightly controlled with CPB, renal autoregulation may be affected, and renal tissue perfusion may be reduced during cardiac surgery. For all of these reasons, ischemia-reperfusion experimental models have been used to study CSA-AKI. Heme toxicity refers to the generation of free heme proteins, which have pathological effects. Recent studies in diverse fields, including sepsis, acute lung injury, hemorrhagic shock, and myocardial infarction, have implicated free heme proteins in the pathogenesis of these diseases. CPB involves removal, oxygenation, propulsion, and return of about 4 L/min of blood volume. This process is known to cause RBC hemolysis. Plasma-free hemoglobin levels are known to be elevated in patients undergoing CPB compared with healthy control subjects. Furthermore, among patients undergoing cardiac surgery, the degree of rise in plasma-free hemoglobin immediately following CPB is predictive of CSA-AKI.^1^ Last, cardiac surgery is a potent inflammatory stimulus, both from the surgery itself and from the effects of the CPB pump. Interleukin-6 is induced by cardiac surgery, and higher levels of interleukin-6 after cardiac surgery have been associated with CSA-AKI. Despite these prior observations, there is no treatment that can reduce CSA-AKI, and management of CSA-AKI is largely supportive or reactionary. There is a strong need for further understanding of the pathogenesis of CSA-AKI and for specific treatments that can prevent or mitigate CSA-AKI.

In this issue of *JACC: Basic to Translational Science*, Vourc’h et al^2^ have investigated an additional hypothesis for the pathogenesis of CSA-AKI. The authors hypothesized that RBC transfusions contain inflammatory proteins that could be responsible for CSA-AKI. Although potentially lifesaving in the treatment of major bleeding or severe anemia, RBC transfusion is by no means a benign intervention. RBC transfusion has been associated with AKI in multiple disease states and after cardiac surgery. RBC transfusion is also a very common practice during and after cardiac surgery. The study by Vourc’h et al^2^ has 2 major phases: a clinical research phase and an experimental biology phase. In the clinical research phase, the authors enrolled a prospective cohort of subjects undergoing cardiac surgery who received RBC transfusion. They obtained 1-mL samples from each RBC transfusion administered to subjects, then centrifuged these samples and stored the supernatant to create a biospecimen bank. They also determined the rate of CSA-AKI, which was 14.7% in their cohort. Next, they measured a panel of proteins from the RBC transfusion biospecimens to identify which proteins present within the RBC transfusions were associated with the development of CSA-AKI. A total of 3 proteins in the RBC transfusions were associated with CSA-AKI (HSP-70, RANTES, and myeloid-related protein[MRP]-14). In a multivariable model, only MRP-14 was independently associated with CSA-AKI.

In the next phase of this study, the authors performed a series of experiments to determine whether MRP-14 can induce AKI. To model the multifactorial pathogenesis of CSA-AKI, the authors performed ischemia-reperfusion (IR) injury in mice by clamping the left renal pedicle for 30 minutes and then reperfusing. The experimental design involved comparing 4 groups: sham surgery alone, sham surgery with MRP-14, IR alone, and combination of IR with MRP-14. The combination of IR and MRP-14 induced the worst renal injury, as assessed by histological analysis at 48 hours after surgery. Furthermore, the combination of IR and MRP-14 induced higher levels of renal monocyte chemoattractant protein-1, a known inflammatory biomarker of AKI, and higher levels of neutrophil recruitment. Overall, the experimental studies demonstrate that exogenous MRP-14 worsens AKI in the setting of IR and has important inflammatory effects in the kidneys including recruitment of neutrophils.

Interestingly, MRP-14 has previously been implicated in the pathogenesis of cardiovascular and renal diseases. MRP-14 (also known as S100A9) is present in neutrophils, macrophages, and platelets, with particularly high abundance in neutrophils. MRP-14 exists largely as a heterodimer (MRP 8/14, also known as S100A8/A9) in the blood; this heterodimer is also called calprotectin, which is a biomarker of neutrophil-associated inflammation.^3^ MRP-14 is known to activate Toll-like receptor 4, a key regulator of inflammation and alarmin response. Platelet levels of MRP-14 have been associated with risk of myocardial infarction. Experimental models of vascular injury show decreased injury and inflammation in MRP-14 knockout mice. One small cohort study of cardiac surgery patients found that higher plasma levels of MRP 8/14 after cardiac surgery were associated with AKI.^4^ The study by Vourc’h et al^2^ pushes this field forward by focusing attention on MRP-14 present within RBC transfusions and the implications of this MRP-14 for CSA-AKI.

The work by Vourc’h et al^2^ has several limitations. The panel of biomarkers was chosen from commercially available assays, and it is not known if other proteins would have stronger associations with CSA-AKI. The prospective cohort study enrolled only a small portion of the total cardiac surgeries at this hospital (3.3%). The cohort would be more representative of cardiac surgery patients if the inclusion criteria allowed for broader enrollment. The experimental model is complex, essential a “2-hit” model with the combination of IR and MRP-14. Because unilateral IR does not result in elevated creatinine levels, the authors relied on renal histology to determine the effects of IR and MRP-14. Furthermore, in cardiac surgery patients treated with RBC transfusions, there are endogenous and exogenous sources of MRP-14, namely MRP-14 derived from the patient’s own neutrophils, macrophages, and platelets vs exogenous MRP-14 derived from RBC transfusions. From the work described, it is difficult to determine the relative proportion and clinical significance of each of these sources. Despite, these limitations, the authors original work is innovative and informs us about a new target in CSA-AKI pathophysiology.

Overall, the authors should be applauded for performing a true translational research study. This worked required collaboration among physicians, surgeons, scientists, and physician-scientists. It required multiyear planning, including the creation of a prospective clinical cohort study and biospecimen bank. It also required detailed experimental work to determine if MRP-14, either alone, or in combination with IR, could induce AKI. This meticulous and multidisciplinary work has brought us 1 step closer to understanding and potentially treating CSA-AKI. As with all good science, this work raises many important questions. Can RBC transfusions be treated or purified to remove deleterious proteins? Where does the MRP-14 in RBC transfusions come from? Is it primarily neutrophil or platelet derived, and does it exist within extracellular vesicles? Would an MRP-14 antagonist mitigate CSA-AKI? More generally, this work is a testament to how little about RBC transfusion we really understand, because we are only recently, more than 100 years after Karl Landsteiner’s seminal discovery of ABO blood groups, appreciating potentially toxic proteins within transfusion products.^5^ The work by Vourc’h et al^2^ has opened the door to many future investigations, and hopefully, to new treatments that can improve outcomes in cardiac surgery and transfusion medicine.

## Funding Support and Author Disclosures

The author has reported that he has no relationships relevant to the contents of this paper to disclose.
